# Analysis of the Mutual Information of Channel Phase Observations in Line-of-Sight Scenarios

**DOI:** 10.3390/e25071038

**Published:** 2023-07-10

**Authors:** Maximilian Matthé, Arsenia Chorti

**Affiliations:** 1 Barkhausen Institut gGmbH, Würzburger Str. 46, 01187 Dresden, Germany; arsenia.chorti@ensea.fr; 2 ETIS UMR 8051, CY Paris University, ENSEA, CNRS, 95000 Cergy, France

**Keywords:** physical layer security, secret key generation, directional statistics, von-Mises distribution

## Abstract

The mutual information of the observed channel phase between devices can serve as an entropy source for secret key generation in line-of-sight scenarios. However, so far only simulated and numeric results were available. This paper derives the probability distribution of the channel phase and corresponding expressions for the mutual information. Moreover, the orientation distribution is optimized in order to maximize the mutual information. All presented results are validated numerically. These outcomes serve as a basis for further analytic investigations on the secret key generation rate and subsequent physical layer security performance analysis in line-of-sight scenarios, such as those encountered in drone-aided communications.

## 1. Introduction

Integrated ground–air–space global networks will rely heavily on the use of multi-hop wireless networking, e.g., using drones, in order to expand the performance and capabilities of future generations of wireless systems. In order to deliver the promised performance, various challenges need to be addressed, e.g., meeting aggressive latency requirements, enabling massive connectivity with low energy consumption and computational effort, jointly with the provision of explicit security guarantees. Another crucial concern arises from the widespread deployment of low-end Internet of Things (IoT) devices. These devices, often manufactured through non-uniform production processes and expected to remain operational for over 10 years, raise important questions about future security architectures. Furthermore, the extensive utilization of artificial intelligence (AI), machine learning (ML), and quantum computing advancements will increase the vulnerability of 6G systems to attacks.

Today’s security architecture of connected devices mainly relies on public key infrastructure (PKI) [1] for authentication and key distribution. Such architecture has proven reliable and useful for a vast range of applications and has proven instrumental in securing both the core network as well as 5G networks incorporating TLS-based protocols. However, upcoming quantum computers are said to on one hand be able to break standard public key-encryption-based handshakes, while on the other hand post-quantum-based asymmetric cryptography can still be computationally expensive for simple, low-end IoT devices such as wireless sensors [2]. Hence, alternative methods for authentication and key distribution must be found.

Here, Physical layer security (PLS) can be a promising candidate to overcome this key distribution problem [3]. In particular, applying secret-key generation (SKG) algorithms that extract the keys from the shared physical channel between the communicating devices [4] presents a light-weight alternative to the complex logistics of PKI, especially when billions of small embedded IoT devices are deployed. In many 5G and 6G scenarios with wide-band communication, the dynamic frequency selectivity of the wireless channel between the pairing devices can be used as a common source of entropy to generate a common secret key [5]. Unfortunately, in static line-of-sight (LOS) scenarios such as those encountered in drone-enabled multi-hop wireless networks, the wireless channels become frequency flat and less dynamic and hence another source of entropy needs to be found.

Despite these difficulties, the use of SKG in LOS conditions can be important for use cases such as drone communications and is motivated by many factors:

- Drones are expected to have widespread use in applications including surveillance, delivery services, and critical infrastructure inspections. These applications often involve the exchange of sensitive and often confidential data that need to be secure against unauthorized access. SKG emerges thus as a viable option for the ad hoc generation of secret keys between drones and critical infrastructure. These can be used in hybrid PLS-crypto systems to establish secure communication channels, ensuring that the transmitted information remains confidential and protected from eavesdropping, while respecting very strict delay constraints by avoiding conventional PKI-based key distribution.
- Secondly, drones operate in wireless environments that are susceptible to other types of malicious attacks, e.g., jamming, spoofing and tampering. Recent works on SKG robustness against such attacks provide the grounds for using such technologies in a trustworthy manner. We note in passing that jamming attacks have been analyzed in [6], tampering during pilot exchange in [7], spoofing during side information exchange in [8], while generalized channel probing has been covered in [9].
- Additionally, drones often operate in dynamic and unpredictable environments, making them vulnerable to both physical and cyber attacks. SKG provides an infrastructureless setting for enabling secure communications in such demanding scenarios.
- Moreover, SKG can lend itself to zero-touch solutions, especially when combined with location and RF-fingerprint-based authentication protocols. Protocols building on these PLS solutions have been proposed for fast authentication of IoT devices [8].

In summary, the use of SKG in drone communications can be instrumental to protect sensitive data, secure wireless communication channels, mitigate risks of physical and cyber attacks, and enable secure interactions within the IoT ecosystem. It can be used in hybrid PLS-crypto systems to ensure the confidentiality of the transmitted data, enhancing the overall security and reliability of drone operations.

Due to the frequency flatness in LOS scenarios, in [10] the authors proposed to use the phase shift of a LOS multiple-input multiple-output (MIMO) channel as the common randomness between devices. In particular, with small wave lengths, small fluctuations in the position of transmitting and receiving antennas can already have a large effect on the channel phase and hence serve as a source of entropy. Moreover, such small changes in the position are hardly visible to malicious observers and hence the channel phase cannot be predicted. The authors in [10] numerically analyzed the mutual information (MI) I(A;B) between the pairing devices, Alice and Bob. Moreover, they also simulated the conditional MI between Alice and Bob given the observer, Eve, to understand the available key generation rate for a specific geometric constellation.

As the novelty, and in contrast to the work in [10] this paper provides analytic expressions for the MI between Alice and Bob given different counts of antennas and signal-to-noise ratios (SNRs). Analytic results are important in order to understand the underlying characteristics of a system and potentially allow to generalize results beyond scenarios that where simulated numerically. In particular, the results presented in this paper are valid for single- and dual-antenna systems and provide a theoretical reasoning on the behaviour of the MI for different SNRs at Alice and Bob. We show that the results match the numeric simulations. In particular, the contributions of this paper are three-fold:

- We analyze the properties of the noise of the channel phase and provide analytic expressions for the probability density function (PDF) of the channel phase under rotating antennas. Here, we build upon the system model from [10], but introduce a geometric approximation in order to make the expressions mathematically tractable. The obtained PDF serves as a basis for the subsequent analysis of I(A;B).
- We derive a tight upper-bound expression for the MI between Alice and Bob which is valid for different SNRs and antenna constellations. This is particularly novel, as the model under consideration has previously only been studied via numeric simulations.
- Using the derived analytic results we show that the orientation distribution of Alice and Bob can be optimized such that I(A;B) reaches the provided upper bound. The obtained expression of the optimal rotation is a direct consequence of the previously derived expressions and is therefore a direct application of our results.

All results are verified with numeric simulations to corroborate their validity. The shown results can serve as a basis for subsequent performance analysis of LOS SKG algorithms in different scenarios, e.g., in drone-aided communications. The remainder of this paper is structured as follows. Section 2 describes the system model in terms of signal and geometry and derives the PDF for the channel phase between Alice and Bob. Analytic expressions for MI between Alice and Bob are derived in Section 3. Section 4 verifies the analytic results with numeric simulations. Finally, concluding remarks are provided in Section 5.

## 2. System Model

### 2.1. Signal Model

**Figure 1:**
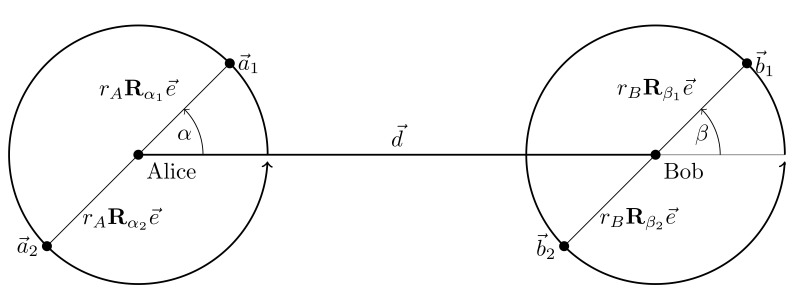
Geometry of the 2D setup.

Consider the two-dimensional model in Figure 1. Alice is located at the origin and Bob’s center is at position $\vec{d}$ and both have multiple antennas, at positions ${\vec{a}}_{i}$ and ${\vec{b}}_{k}$, respectively, where i,k are the antenna indices at Alice and Bob, respectively. From the geometry we see that ${\vec{a}}_{i}=r_{A}R_{\alpha_{i}}\vec{e}$ and ${\vec{b}}_{k}=\vec{d}+r_{B}R_{\beta_{k}}\vec{e}$, where $\alpha_{i}$ and $\beta_{k}$ are the antenna rotations of the *i*-th and *k*-th antenna at Alice and Bob, $R_{\gamma }$ is a rotation matrix that rotates around the angle γ, $r_{A},r_{B}$ are the antenna radius for Alice and Bob, respectively, and $\vec{e}$ is the unit vector pointing towards the right.

Assuming that Alice sends a non-modulated carrier x(t) from antenna *i*, the received signal $y_{ik}\left(t\right)$ at Bob’s *k*th antenna is given by

(1) $$\begin{array}{cc} x(t) & =\exp(j2\pi f_{c}t) \end{array}$$

(2) $$\begin{array}{cc} \;y_{\mathrm{ik}}\left(t\right) & =\exp\left(j2\pi f_{c}\left(t-\frac{d_{\mathrm{ik}}}{c}\right)\right)+{\tilde{n}}_{\mathrm{ik}}\left(t\right) \end{array}$$

(3) $$\begin{array}{cc} \; & =\exp\left(j2\pi f_{c}t\right)\exp\left(j\phi_{ik}\right)+{\tilde{n}}_{ik}\left(t\right) \end{array}$$

where *c* is the speed of light, $f_{c}$ is the carrier frequency and

(4) $$\begin{array}{cc} d_{\mathrm{ik}}= & ∥\vec{d}+{\vec{b}}_{k}-{\vec{a}}_{i}∥ \end{array}$$

is the distance between the *i*th and *k*th antenna and $\phi_{\mathrm{ik}}=d_{\mathrm{ik}}/c\;\mathrm{mod}\;2\pi$ is the phase of the channel between these antennas (Note that in reality, real-valued signals are transmitted and received. However, for the mathematical presentation, the complex passband representation is more compact.). ${\tilde{n}}_{\mathrm{ik}}\left(t\right)$ is AWGN. Letting Bob employ an ideally synchronized matched filter receiver which integrates the received signal over a period of the carrier frequency we have

(5) $$\begin{array}{cc} \;y_{B,\mathrm{ik}} & =\int_{0}^{1/f_{c}}y_{B,ik}\left(t\right)\exp\left(-j2\pi f_{c}t\right)\mathrm{dt} \end{array}$$

(6) $$\begin{array}{cc} \; & =\frac{1}{f_{c}}\exp\left(j\phi_{\mathrm{ik}}\right)+{\tilde{n}}_{B,\mathrm{ik}} \end{array}$$

There, ${\tilde{n}}_{B,\mathrm{ik}}$ is once again complex AWGN with variance $\sigma_{B}^{2}$ since the integration happens over a full period of the carrier. Without loss of generality we assume $f_{c}=1$ and hence we define the SNR at Bob by ${\mathrm{SNR}}_{B}=1/\sigma_{B}^{2}$. A similar consideration can be performed for signals transmitted from Bob to Alice. In particular, we note that the phase between antennas *i* and *k* is reciprocal, meaning that the channel phase from Alice to Bob is equal to the phase from Bob to Alice.

**Figure 2:**
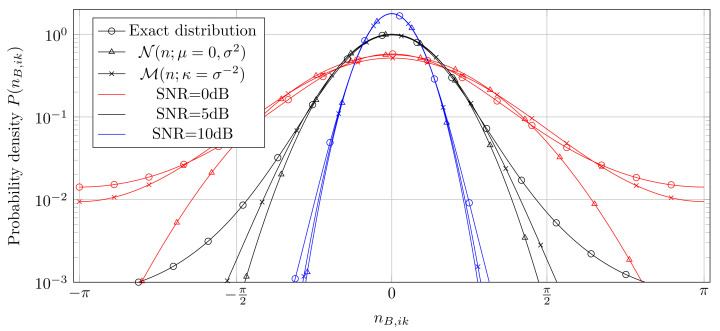
Probability distribution of the real phase error $n_{B,ik}$ and approximations by Gaussian and von-Mises distributions for different SNR. For high SNR, both Gaussian and von-Mises match the exact distribution well, whereas for low SNR, the von-Mises distribution is advantageous.

Now, to estimate the phase of the channel, Bob takes the angle of the matched filter output, yielding

(7) $$\begin{array}{cc} {\hat{\phi }}_{B,\mathrm{ik}} & =\arg\left(y_{B,\mathrm{ik}}\right)=\phi_{\mathrm{ik}}+n_{B,\mathrm{ik}}, \end{array}$$

where $n_{B,\mathrm{ik}}$ is the measurement noise which is not Gaussian distributed anymore. Instead, (7) describes the phase of the non-zero mean complex Gaussian random variable $y_{B,\mathrm{ik}}∼\mathrm{CN}\left(e^{j\phi_{\mathrm{ik}}},\sigma_{B}^{2}\right)$. The distribution of this phase has been thoroughly analyzed in [11] and the authors have shown that for high SNR the distribution matches a Gaussian distribution as $n_{B,\mathrm{ik}}∼N\left(0,\frac{\sigma^{2}}{2}\right)$. In fact, for high SNR the periodic von-Mises distribution [12] $y_{B,\mathrm{ik}}∼M\left(\kappa \right)=\frac{1}{2\pi I_{0}\left(\kappa \right)}\exp\left(\kappa \cos\left(\phi \right)\right)$ with $\kappa =2\sigma_{B}^{-2}$ matches as well and provides the avantageous property of being periodic with 2π. For lower SNR, both the Gaussian and the von-Mises distribution do not fully reflect the original distribution, however the von-Mises distribution better follows the longer tail of the actual distribution, see Figure 2 for an illustration. Therefore, in the sequel we assume $n_{B,\mathrm{ik}}$ to be von-Mises distributed and we will point out the numerical consequences later on. Note that analogous considerations can be done for the measurements at Alice, yielding the same results, which we omit here for brevity.

### 2.2. Geometry Model

The channel phase between Alice and Bob for varying α,β is given by

(8) $$\begin{array}{cc} \phi_{\mathrm{ik}} & =2\pi \frac{d_{\mathrm{ik}}}{\lambda }\;\mathrm{mod}\;2\pi \end{array}$$

where $\lambda =cf_{c}$ is the carrier’s wavelength. Using (4) we get

(9) $$\begin{array}{cc} d_{\mathrm{ik}} & =\sqrt{{\left(d-r_{A}\cos\alpha_{i}+r_{B}\cos\beta_{k}\right)}^{2}+{\left(r_{A}\sin\alpha_{i}-r_{B}\sin\beta_{k}\right)}^{2}} \end{array}$$

Assuming that $d\gg r_{A}$ and $d\gg r_{B}$ we omit the second part under the square root and find

(10) $$\begin{array}{ccc} \;\phi_{\mathrm{ik}} & \approx 2\pi \frac{d}{\lambda }-2\pi \frac{r_{A}\cos\alpha_{i}}{\lambda }+2\pi \frac{r_{B}\cos\beta_{i}}{\lambda } & \mathrm{mod}\;2\pi \end{array}$$

(11) $$\begin{array}{ccc} \; & =\phi_{0}+\phi_{A,i}+\phi_{B,k} & \;\mathrm{mod}\;2\pi . \end{array}$$

Here, $\phi_{0}$ is a constant due to the average distance and $\phi_{A,i},\phi_{B,k}$ describe the phase contributions due to the rotation of Alice and Bob.

Let us assume $\alpha_{i}$ and $\beta_{k}$ are uniformly distributed, i.e., $P\left(\beta_{k}\right)={\left(2\pi \right)}^{-1}$ and Alice and Bob have no preferred orientation. Then, applying random variable transformation to $\phi (\beta_{k})$ yields

(12) $$\begin{array}{cc} \phi_{B,k}\left(\beta_{k}\right) & =2\pi \frac{r_{B}\cos\left(\beta_{k}\right)}{\lambda }\;\mathrm{mod}\;2\pi \end{array}$$

(13) $$\begin{array}{cc} \; & =2\pi \frac{r_{B}\cos\left(\beta_{k}\right)}{\lambda }+n\cdot 2\pi \;s.t.\;\phi_{B,k}\in \left[-\pi ,\pi \right] \end{array}$$

(14) $$\begin{array}{cc} \beta_{k}\left(\phi_{B,k}\right) & \;=\cos^{-1}\left(\frac{\lambda \cdot \phi_{B,k}}{2\pi r_{B}}-\frac{\lambda \cdot n}{r_{B}}\right) \end{array}$$

(15) $$\begin{array}{cc} \frac{d\beta_{k}\left(\phi_{B,k}\right)}{d\phi_{B,k}} & =\frac{\lambda }{2\pi }\frac{1}{\sqrt{1-{\left(\frac{\lambda \cdot \phi_{B,k}}{2\pi r_{B}}-\frac{\lambda \cdot n}{r}\right)}^{2}}} \end{array}$$

and thus

(16) $$\begin{array}{cc} P_{\phi_{B,k}}\left(\phi_{B,k}\right) & =P_{\beta }\left(\beta_{k}\left(\phi_{B,k}\right)\right)\frac{d\beta_{k}\left(\phi_{B,k}\right)}{d\phi_{B,k}} \end{array}$$

(17) $$\begin{array}{cc} \; & =\frac{\lambda }{2\pi^{2}r_{B}}\sum_{n}\frac{1}{\sqrt{1-{\left(\frac{\lambda (\phi_{B}-2\pi n)}{2\pi r_{B}}\right)}^{2}}}, \end{array}$$

where the summation is over all *n* such that the square root is real. In particular, for $r_{B}\leq \lambda /2$ the expression simplifies to

(18) $$\begin{array}{cc} P_{\phi_{B,k}}\left(\phi_{B,k}\right) & =\frac{1}{\pi^{2}\sqrt{1-{\left(\frac{\lambda \cdot \phi_{B,k}}{2\pi r_{B}}\right)}^{2}}}, \end{array}$$

where for $r_{B}<\lambda /2$ not even all channel phases are reached.

On the other hand, taking the limit for infinite $r_{B}$ we obtain

(19) $$\begin{array}{cc} \lim_{r_{B}\to \infty }P_{\phi_{B,k}}\left(\phi \right) & =\frac{1}{2\pi^{2}U}\sum_{n}\frac{1}{\sqrt{1-{\left(\frac{k}{U}\right)}^{2}}} \end{array}$$

(20) $$\begin{array}{cc} \; & =\frac{1}{2\pi^{2}U}\int_{-1}^{1}\frac{\mathrm{Udx}}{\sqrt{1-x^{2}}}=\frac{1}{2\pi }, \end{array}$$

where $U=\frac{r_{B}}{\lambda }$ and $\mathrm{dx}=\frac{n}{U}-\frac{n+1}{U}=\frac{1}{U}$ and hence for large $r_{B}$ the distribution approaches uniformity.

**Figure 3:**
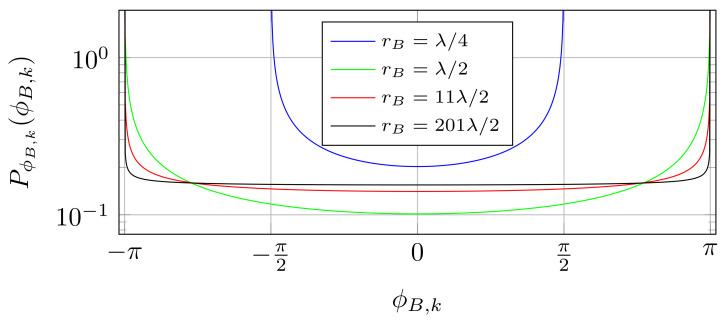
PDF of $\phi_{B,k}$ for different antenna rotation radii. With increasing radius, the PDF approaches the uniform distribution.

Figure 3 shows the phase distribution for different ratios of $\lambda /r_{B}$. As visible, for $\lambda /r_{B}=\frac{1}{4}$, the channel phase does not reach the entire range of angles, because the antenna radius is too small. On the other hand, the bigger the $r_{B}$, the more the distribution approaches a uniform distribution, and already for $\lambda /r_{B}=\frac{1}{2}$ the real distribution is very close to uniform. Note that analogous considerations can be carried for the distribution of $\phi_{A,i}$, yielding the analog expressions.

## 3. Mutual Information

For calculating the mutual information I(A;B) we assume $\phi_{0}=0$, since a constant angle offset does not influence the mutual information between Alice and Bob. Moreover, in the sequel we assume that if Alice or Bob have two antennas, then $\alpha_{1}=\alpha_{2}+\pi =\alpha$ and $\beta_{1}=\beta_{2}+\pi =\beta$, i.e., the antennas at both Alice and Bob have an angle offset of 180 degree. In this case, $\phi_{A,1}=-\phi_{A,2}$ and $\phi_{B,1}=-\phi_{B,2}$.

### 3.1. Single-Antenna Case

In the most basic 1×1-case, where Alice and Bob have one antenna each and only Bob rotates their antenna, the measurement model can be written as

(21) $$\begin{array}{cccc} {\hat{\phi }}_{A,11} & =\phi_{B,1}+n_{A,11} & \;{\hat{\phi }}_{B,11} & =\phi_{B,1}+n_{B,11}, \end{array}$$

where both summations are performed mod2π. Here, we consider $\phi_{A,0}=0$ since $\phi_{A,0}$ is constant and any constant angle will not influence I(A;B). We substract both equations and get

(22) $$\begin{array}{cc} {\hat{\phi }}_{A,11} & ={\hat{\phi }}_{B,11}+n_{B,11}-n_{A,11}, \end{array}$$

and therefore

(23) $$\begin{array}{cc} I({\hat{\phi }}_{A,11};{\hat{\phi }}_{B,11}) & =I({\hat{\phi }}_{B,11}+n;{\hat{\phi }}_{B,11}) \end{array}$$

(24) $$\begin{array}{cc} \; & =H\left({\hat{\phi }}_{B,11}+n\right)-H\left({\hat{\phi }}_{B,11}+n|{\hat{\phi }}_{B,11}\right) \end{array}$$

(25) $$\begin{array}{cc} \; & =H\left(\phi_{B,1}+n_{A,11}\right)-H\left(n\right), \end{array}$$

where $n=n_{B,11}-n_{A,11}$ is the sum of the measurement noise at Alice’s and Bob’s antenna.

In (17) it was shown that the distribution of $\phi_{B,1}$ is already nearly uniform. Since additive noise results equalizes the distribution more, we assume ${\hat{\phi }}_{A,11}$ to be nearly uniformly distributed. Therefore, the first component of the mutual information can be approximated (and upper bounded) by the entropy of the uniform distribution on [−π,π):

(26) $$\begin{array}{c} H\left(\phi_{B,1}+n_{A,11}\right)⪅\log_{2}\left(2\pi \right) \end{array}$$

For the second part, $H(n_{B,11}-n_{A,11})$ we consider that both $n_{A,11},n_{B,11}$ are independent and, for higher SNR, nearly Gaussian distributed. Hence, the difference of both is also distributed according to a Gaussian with variance $\mathrm{Var}\left(n\right)=\sigma_{A}^{2}+\sigma_{B}^{2}$. However, before we saw that the periodice von-Mises distribution approximates the real phase distribution better for lower SNRs, and therefore we assume that *n* is von-Mises distributed with

(27) $$\begin{array}{c} n∼M(\kappa =\frac{2}{\sigma_{A}^{2}+\sigma_{B}^{2}}). \end{array}$$

Note, that this expression does not hold exactly for lower SNR, since in this case the variances do not add completely due to the summation mod2π. The deviation will be pointed out in the simulation results. Hence, $I({\hat{\phi }}_{A,11};{\hat{\phi }}_{B,11})$ can be calculated by

(28) $$\begin{array}{cc} I({\hat{\phi }}_{A,11};{\hat{\phi }}_{B,11}) & \leq \log_{2}\left(2\pi \right)-H_{M}\left(\kappa =\frac{2}{\sigma_{A}^{2}+\sigma_{B}^{2}}\right) \end{array}$$

(29) $$\begin{array}{cc} \; & ={\left.\frac{1}{\ln2}\frac{\kappa I_{1}\left(\kappa \right)}{I_{0}\left(\kappa \right)}-\log_{2}\left(I_{0}\left(\kappa \right)\right)\right|}_{\kappa =\frac{2}{\sigma_{A}^{2}+\sigma_{B}^{2}}} \end{array}$$

(30) $$\begin{array}{cc} & =:I_{1\times 1}\left(\sigma_{A}^{2},\sigma_{B}^{2}\right),\; \end{array}$$

where $H_{M}\left(\kappa \right)$ is the differential entropy of a von-Mises distributed random variable with parameter κ.

### 3.2. 1×2 System

The case with multiple antennas can be directly derived from the fundamental single-antenna case. First, we consider a 1×2 system where Bob has 2 antennas, and Alice has 1 antenna, and only Bob rotates their antennas, the measurement equations become:

(31) $$\begin{array}{cccc} {\hat{\phi }}_{A,11} & =\phi_{B,1}+n_{A,11}, & \;{\hat{\phi }}_{B,11} & =\phi_{B,1}+n_{B,11}, \end{array}$$

(32) $$\begin{array}{cccc} {\hat{\phi }}_{A,12} & =-\phi_{B,1}+n_{B,12}, & \;{\hat{\phi }}_{B,12} & =-\phi_{B,1}+n_{B,12}. \end{array}$$

Essentially, now Bob and Alice have two independent noisy observations of the same underlying random variable $\phi_{B,1}$. Therefore, the mutual information can be calculated from the fundamental case by a simple SNR shift:

(33) $$\begin{array}{cc} I_{1\times 2}\left(\sigma_{A}^{2},\sigma_{B}^{2}\right) & :=I({\hat{\phi }}_{A,11},{\hat{\phi }}_{A,12};{\hat{\phi }}_{B,11},{\hat{\phi }}_{B,12}) \end{array}$$

(34) $$\begin{array}{cc} & =I_{1\times 1}\left(\frac{\sigma_{A}^{2}}{2},\frac{\sigma_{B}^{2}}{2}\right). \end{array}$$

### 3.3. 2×2 System

Eventually, the 2×2-case where both Alice and Bob have two antennas each, and both Alice and Bob rotate their antennas yields the following measurement equations for Alice,

(35) $$\begin{array}{cc} {\hat{\phi }}_{A,11} & =\phi_{A,1}+\phi_{B,1}+n_{A,11} \end{array}$$

(36) $$\begin{array}{cc} {\hat{\phi }}_{A,12} & =\phi_{A,1}-\phi_{B,1}+n_{A,12} \end{array}$$

(37) $$\begin{array}{cc} {\hat{\phi }}_{A,21} & =-\phi_{A,1}+\phi_{B,1}+n_{A,21} \end{array}$$

(38) $$\begin{array}{cc} {\hat{\phi }}_{A,22} & =-\phi_{A,1}-\phi_{B,1}+n_{A,22}, \end{array}$$

and similar equations can be formulated for Bob’s reception. Compared to the case where only Bob rotated their antennas, the system now has two degrees of freedom, namely $\phi_{A,1}$ and $\phi_{B,1}$. Therefore, we expect the mutual information to be double compared to the case with a single degree of freedom. At the same time, each side has four measurements to estimate two parameters. Compared to the single-antenna case, where each side had one measurement to estimate one parameter, the SNR is again doubled. Consequently, the mutual information in the multi-antenna case is given by

(39) $$\begin{array}{cc} I_{2\times 2}\left(\sigma_{A}^{2},\sigma_{B}^{2}\right) & :=I\left(\left(\begin{array}{c} {\hat{\phi }}_{A,11}\\ {\hat{\phi }}_{A,12}\\ {\hat{\phi }}_{A,21}\\ {\hat{\phi }}_{A,22} \end{array}\right);\left(\begin{array}{c} {\hat{\phi }}_{B,11}\\ {\hat{\phi }}_{B,12}\\ {\hat{\phi }}_{B,21}\\ {\hat{\phi }}_{B,22} \end{array}\right)\right) \end{array}$$

(40) $$\begin{array}{cc} \; & =2\cdot T_{1\times 1}\left(\frac{\sigma_{A}^{2}}{2},\frac{\sigma_{B}^{2}}{2}\right). \end{array}$$

### 3.4. Optimal Rotation Distribution

Before, we saw that (25) is maximized, if $\phi_{A,i}$ and $\phi_{B,k}$ are uniformly distributed. Hence, finding a distribution for α,β that yields a uniform channel phase will maximize I(A;B). When Alice and Bob have no preferred orientation and hence α,β are uniformly distributed, ϕ is distributed according to (17). On the other hand, for $r_{B}=\lambda /2$, if

(41) $$\begin{array}{cc} P_{\beta } & =\frac{1}{4}\sqrt{1-\cos^{2}\left(\beta \right)} \end{array}$$

is inserted into (16) it becomes apparent that $\phi_{B,k}$ is uniformly distributed for the given optimal rotation distribution in (41).

## 4. Simulation Results

This section shows simulated MI between Alice and Bob along with the analytic results from above. The results were obtained in Python by simulating (6) and then taking the phase according to (7). We assume ideal synchronization and no hardware impairments like phase noise of the oscillators. The distances $d_{ik}$ that are used to generate $\phi_{ik}=d_{ik}/c$ have been obtained by using the exact geometry expression from (4) for random rotations of the antennas at Alice and Bob. The distribution of the rotation angle of Alice and Bob was uniform except for the optimized rotation distribution described in the previous section. The mutual information was estimated using the Python NPEET package [13]. For each SNR point, sufficiently many samples were obtained until the curves became smooth and the upper bound of calculatable mutual information imposed by the NPEET algorithm [14] was not reached. In particular, this corresponded to 500.000 angle realizations per SNR point. The source code used for obtaining the results is contained in the supplementary material of this paper.

**Figure 4:**
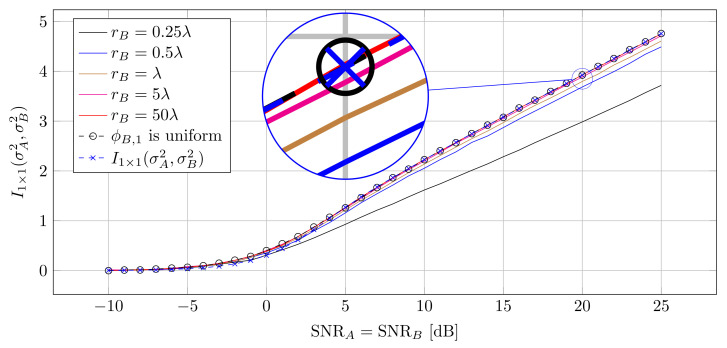
Mutual information for different antenna radius. The higher the antenna radius, the closer the simulated MI approaches the theoretic curve.

Figure 4 shows the I(A;B) for the single-antenna system with different $r_{B}$. The distance between Alice and Bob was set to d=100λ. As mentioned before, with increasing $r_{B}$, the distribution of $\phi_{B,1}$ nears the uniform distribution and the uniform distribution of $\phi_{B,1}$ maximizes the obtained mutual information. Figure 4 also shows the simulated MI curve, when $\phi_{B,1}$ is truly uniformly distributed. For $r_{B}=\lambda$ these curves are reasonably close and they virtually overlap for $r_{B}=5\lambda$. Moreover, we show that for SNR > 5 dB the curve for uniform $\phi_{B,1}$ overlaps the theoretic curve $I_{1\times 1}$.

**Figure 5:**
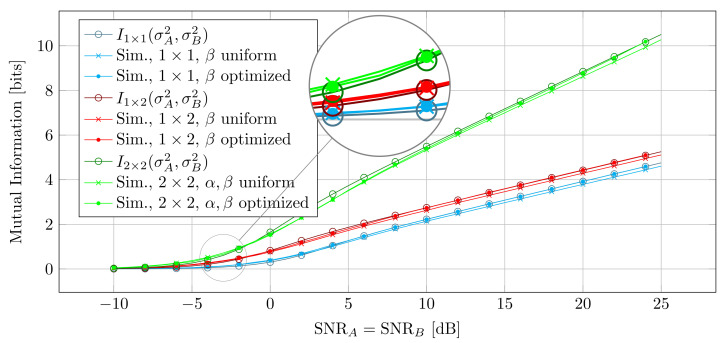
Mutual Information for equal SNR for Alice and Bob.

Figure 5 shows the simulated MI for the single and multi-antenna systems with $r_{B}=\lambda ,d=100\lambda$ along with the theoretic curves. As can be seen, for SNR > 5 dB the simulated curves are closely upper bounded by the theory, whereas for the lower SNR the theoretic curves estimate the mutual information too low. This is due to the fact that in lower SNR $\mathrm{Var}\left(n\right)\neq \sigma_{A}^{2}+\sigma_{B}^{2}$ because the noise samples are added mod2π and their entropy is upper bounded by the uniform distribution. Therefore, H(n) in (25) is estimated too high. However, the approximation is still very close.

**Figure 6:**
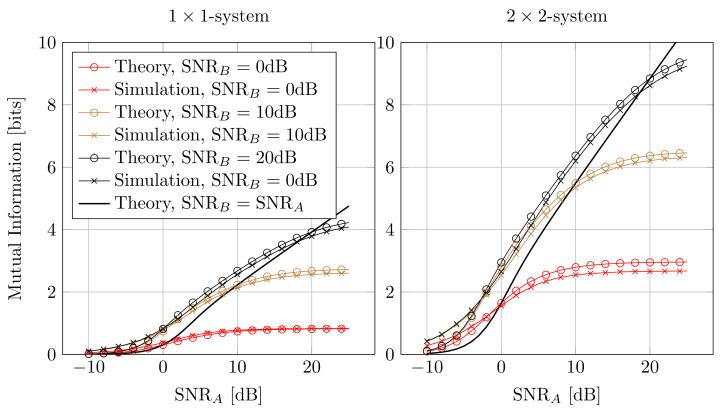
Mutual Information for different SNR for Alice and Bob.

Figure 6 shows the measured and theoretic mutual information for single and multi-antenna systems where the SNR at Alice and Bob is different. This can, e.g., happen in a non-symmetric scenario like in an up- and downlink of a celluar system. Naturally, when the SNR of Bob is fixed, I(A;B) approaches a finite limit for high ${\mathrm{SNR}}_{A}$. The theoretic and simulated curves match well, and still the theoretic curve is an upper bound for I(A;B) for SNR>5dB.

Figure 5 also shows the simulated curves for I(A;B) when Alice and Bob distribute their orientation according to (41). As can be seen, in this case I(A;B) is above that of a uniform orientation of Alice and Bob. Moreover, the simulated curves overlap the curves for the analytic upper bound and hence prove that the MI between Alice and Bob indeed has been maximized.

## 5. Conclusions

In this paper, we derive analytic expressions for the mutual information between two communicating devices, where the shared information is the phase of the reciprocal line-of-sight MIMO channel between both devices. Using randomly moving antennas, the channel phase becomes a random variable that is estimated at both sides. Such commonly estimated information can be used for example as an entropy source for key-generation in physical-layer-based security schemes.

The derived expressions match well with the simulation results, proving their validity. The results can be calculated much faster and more flexibly compared to running tedious numerical simulations for different SNR combinations. Moreover, we derived an antenna rotation scheme which maximizes the mutual information and proved the validity with simulation results. The results are valid for one or two transmit antennas only, and cannot be straightforwardly extended to four or more antennas. Another open point is the extension of the geometry to the three-dimensional space, where Alice and Bob have two degrees of freedom in their rotation, respectively.

The obtained results indicate that the channel phase of an LOS MIMO channel contains sufficient information to synchronously generate random keys. At a high SNR, which can be well assumed in LOS scenarios, each antenna can provide up to five bits of entropy per measurement under ideal conditions. The obtained results help engineers estimate the required SNR for real-world experiments and the presented derivations enable researchers to elaborate on more sophisticated system models including higher dimensions and more antennas.

### Future Works

In future works, the presented results shall be verified with real-world measurements to validate the obtained expressions. Here, a solution using software-defined radios and offline signal processing shall be the first step to compare theoretic and simulated results with real-world measurements.

On the theoretical side, it is of utmost importance to derive expressions when an eavesdropper “Eve” enters the stage, because a secret key generation algorithm needs to not only consider common information between Alice and Bob but also what information is available to an eavesdropper [4]. Here, the position of Eve relative to Alice and Bob will have a major influence on the dropped information, because, as this paper has shown, some rotation angles exhibit more information than others. Hence, expressions involving the position of Alice, Bob and Eve need to be developed.

Finally, an actual key generation algorithm that exploits the channel phase between devices should be designed, theoretically and numerically evaluated and eventually validated with real measurements. Here, a multi-step approach as described in [15] consisting of Randomness Extraction, Quantization, Information Reconciliation and Privacy Amplication can be a promising solution.

PLSPhysical layer security SKGsecret-key generation IoTInternet of Things PKIpublic key infrastructure LOSline-of-sight SNRsignal-to-noise ratio MImutual information PDFprobability density function MIMOmultiple-input multiple-output

## Data Availability

Source code to regenerate the simulation results is provided as supplementary material to this paper.

## Supplementary Materials

The following supporting information can be downloaded at: https://www.mdpi.com/article/10.3390/e25071038/s1, Source code for regenerating the simulation results.
